# Precision Molding Simulation Study of 3D Ultra-Thin Glass Components for Smartwatches

**DOI:** 10.3390/mi16050584

**Published:** 2025-05-16

**Authors:** Xinfeng Zhao, Shunchang Hu, Peiyan Sun, Wuyi Ming

**Affiliations:** 1 College of Water Conservancy Engineering, Yellow River Conservancy Technical University, Kaifeng 475000, China; zhaoxinfeng@yrcti.edu.cn; 2 Henan Key Laboratory of Intelligent Manufacturing of Mechanical Equipment, Zhengzhou University of Light Industry, Zhengzhou 450002, China; peiyansun252@163.com; 3 Guangdong Provincial Key Laboratory of Digital Manufacturing Equipment, Guangdong Huazhong University of Science and Technology Industrial Technology Research Institute, Dongguan 523808, China

**Keywords:** 3D ultra-thin glass components, forming quality, optimization scheme, forming pressure, forming temperature

## Abstract

High stress and shape deviation during the glass forming process often led to low yield rates, posing a challenge in the production of high-precision smartwatch components. To address this issue, a numerical model was developed to simulate and analyze the forming behavior of 3D curved glass. The study focused on achieving a balance between energy consumption and key quality attributes, such as residual stress and shape accuracy. Results showed that forming pressure primarily affects shape deviation, while forming temperature plays a dominant role in energy usage and residual stress. Through orthogonal experiments, optimal parameters were identified: a forming temperature of 630 °C, pressure of 0.25 MPa, and cooling rate of 0.25 °C/s effectively minimize residual stress. Meanwhile, shape deviation is minimized at 630 °C, 0.30 MPa, and a cooling rate of 0.75 °C/s. Energy efficiency analysis indicated that low efficiency occurs at 610 °C with a 3 °C/s heating rate. Furthermore, NSGA-II multi-objective optimization validated the model’s accuracy, with prediction errors under 20%, offering valuable guidance for the precise fabrication of smartwatch glass.

## 1. Introduction

Smartwatches, a crucial component of wearable devices, bridge the gap between conventional timepieces and smartphones by offering a certain level of information processing capability [1,2,3]. Due to continuous technological advancements and intensified market competition, smartwatches have been evolving, providing unprecedented user experiences [4,5]. Manufacturers have been striving for innovation to stand out in the competitive landscape and meet customer demands for higher performance and better user experiences. The distinctive optical quality, transparency, toughness, scratch resistance, and superior weatherability of 3D curved glass [6,7,8], enhances the esthetic appeal of products and is poised to become a key differentiator in the high-end smartwatch market [9].

The glass molding process (GMP) [10,11,12], a precise thermal forming method, is a crucial manufacturing step for the curved screens used in smartwatches. The quality of glass surface creation is directly impacted by the GMP parameters and material properties [13,14]. GMP stands out for its high forming precision, short manufacturing cycles, low cost, and lack of pollution, making it ideal for mass production of optical components such as spherical/aspherical glass lenses and freeform optical elements [15]. Moreover, the thermal bending process itself can be combined with advanced surface functionalization techniques to further enhance the comprehensive properties of glass [16]. Numerous academics have studied glass molding in great detail. For example, Tao and Yuan [17] confirmed that the material parameters dictate the size of residual stresses by examining the impacts of specific heat capacity and thermal expansion coefficient on residual stress prediction in molded glass. Yan et al. [18] proposed a two-step pressing process based on the nonlinear thermal expansion properties of glass. The heat transfer phenomenon was simulated by considering the dependence of specific heat and thermal conductivity on temperature. The shortest heating time and pressure changes were successfully predicted using a numerical model and observed glass characteristics. Su et al. [19] utilized finite element methods to simulate and predict variations in group refractive index, thereby providing improved geometric designs for desired lenses. Finite element-assisted compensation processes can be employed to predict the final optical performance of molded glass components.

Molding process parameters significantly influence the processing quality of glass components. Constructing accurate predictive models and optimizing these process parameters accordingly holds important value for significantly improving product quality [20]. Recent research has focused on delving deeper into the inherent relationship between processing quality and process parameters, along with their optimization strategies. For example, Zhang et al. [21] explored the sustainability of ultra-thin GMP, revealing the effects of different process parameters on energy efficiency. Ming et al. [22] thoroughly compared the equipment implementation and the GMP apparatus as a whole, and a thorough description of the latest theoretical advancements in the use of ultrasonic-assisted technologies and high-frequency microwave in GMP was given. He et al. [23] studied the entire multi-position bending process of curved glass screens for smartphones by combining numerical simulation and experimental methods. The research analyzed the distribution of high-stress occurrences and identified the key factors influencing residual stress and shape deviation in the final products.

Considering the status of research and theoretical underpinnings at the moment, forming temperature, forming pressure, and material characteristic are identified as significant factors influencing the quality of curved glass molding in GMP [24,25]. Moreover, the glass forming process involves substantial energy efficiency, and the pursuit of high-quality glass components inevitably leads to a significant increase in production costs [26]. Research on glass component molding technologies primarily focuses on large and medium-sized glass components, such as automotive glass components [27], glass panels for fingerprint locks [10], and 3D curved screens for smartphones [23]. The 3D curved glass of smartwatches, due to its small size and extremely thin thickness, requires high precision in controlling the forming temperature and pressure. In addition, because hot bending is a high-temperature and high-pressure process, it requires a significant amount of energy to heat the molds and the glass to the required temperature, making energy reduction crucial as well. Studies on small glass components are limited, particularly in the research of molding technologies for smartwatches. Thus, this study developed a numerical model for the forming of smartwatch glass to improve the forming quality and reconcile efficiency of manufacturing with the quality of the final product. The results were experimentally validated, achieving multi-objective optimization that enhanced both product quality and energy efficiency.

## 2. GMP Simulation Modeling

The small size of smartwatches demands high surface precision for their glass components. Furthermore, the heat transfer in the thickness direction is significant, adding complexity to the glass processing control. This study utilized MSC. Marc to numerically simulate the forming process of smartwatch glass components. By observing the whole process of the glass workblank in real-time, a better understanding of the forming mechanisms of smartwatch glass components was achieved.

### 2.1. Geometric Dimensioning

**Figure 1:**
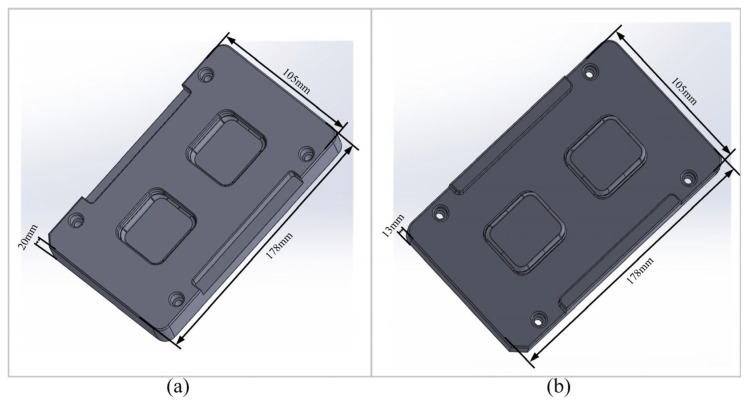
Major dimensions of the mold for 3D glass forming in smartwatches: (**a**) upper mold; (**b**) lower mold.

This study focuses on the 3D curved glass components of smartwatches, meticulously examining the molding process and parameters. Figure 1 illustrates the mold model utilized in this research. The upper mold is designed with precise dimensions of 178 × 105 mm and a height of 20 mm, ensuring an accurate fit and optimal molding conditions. Similarly, the lower mold measures 178 × 105 mm with a height of 13 mm, providing a complementary structure for the molding process.

### 2.2. Material Property

G-11 glass was chosen for simulation analysis in this study, setting the reference temperature at 618 °C [28,29], consistent with its actual processing parameters. Table 1 details the mechanical and thermal parameters of graphite material used to fabricate the molds, which is widely employed in the production of glass component molds due to its unique chemical and physical stability, along with its ability to maintain stable performance at temperatures between from 800 and 1000 °C. During the heating stage, the viscosity variation curve of the glass was fitted using the William–Landel–Ferry model. Additionally, the Narayanaswamy model was employed to elucidate the intrinsic relationship between temperature and structural relaxation during the annealing and cooling phases [30,31]. The heat transfer coefficient between nitrogen and the mold was denoted as *h*_N_, while the coefficient between glass and the mold was denoted as *h*_M_. Based on the referenced literature, *h*_N_ was set to 20 W/(m^2^·K) and *h*_M_ to 2800 W/(m^2^·K) [32,33]. Table 2 provides specific information on the glass’s structural and stress relaxation properties. The William–Landel–Ferry data were set to 569 °C, where the constants C_1_ and C_2_ were taken as 12.41 and 129.0, respectively [34].

### 2.3. Boundary Conditions

In this study, the temperature of both the mold and the glass was set to 25 °C (room temperature), and this served as the baseline for constructing the simulation model’s initial conditions. Based on actual operating conditions, the model’s load conditions and boundary conditions were established, including restrictions on the x, y, and z directions of movement for the lower mold and the x and y directions for the upper mold, with the upper mold set to move along the negative *z*-axis direction under specific pressure conditions.

**Figure 2:**
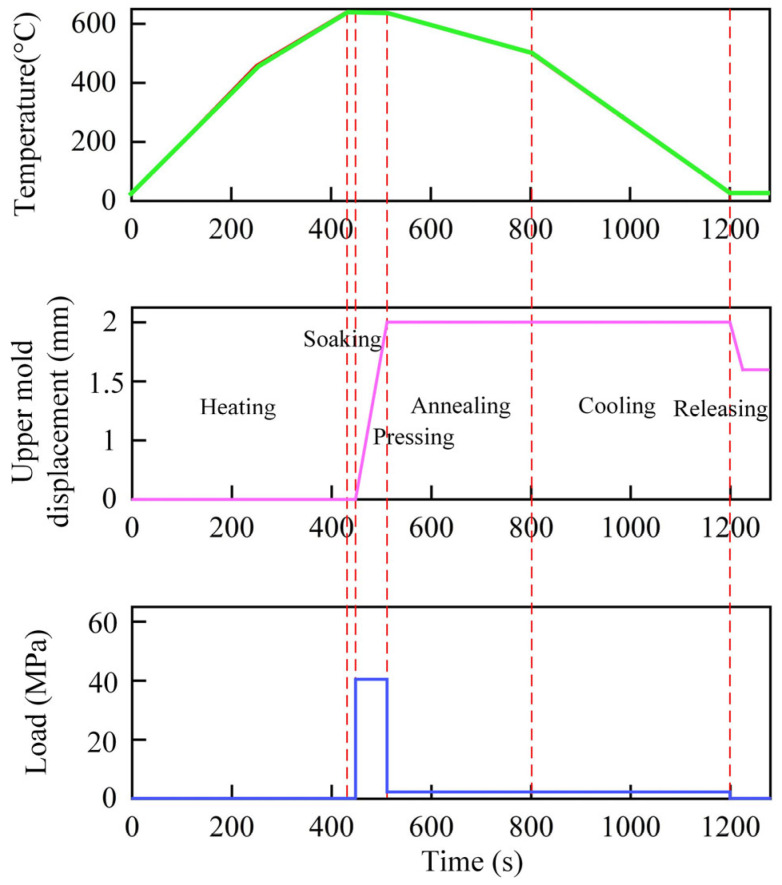
Boundary conditions at various stages of the glass molding process.

Heating, holding, and cooling were the three primary phases of the temperature management approach for the glass-forming process. With a steady heating rate of 1.5 °C/s, the complete heating and soaking procedure should take around 430 s. During the forming stage, the glass component’s upper mold smoothly moved 10 mm along the negative z direction, successfully completing the glass molding process. This process is anticipated to take 80 s to ensure the glass achieves the desired shape, after which the glass component and the mold will enter a slow annealing phase to reduce the temperature to approximately 500 °C and alleviate internal stress within the glass. After that, the temperature will rapidly decrease to 25 °C to guarantee the glass component’s stability and longevity. Applying a steady stress of around 400 N to the mold’s upper surface was a crucial step in this process because it is necessary to prevent variations in the glass during the annealing and cooling stages, thus ensuring it maintains the intended shape and accuracy. The setting of the boundary conditions is shown in Figure 2. In order to replicate the contact behavior between the glass and the mold, a stick-slip friction model was also used in the simulation. Key parameters for this model include the relative displacement transition zone from sliding to stick friction, friction coefficient multiplier, friction tolerance, and friction coefficient, set to 0.1, 1.05, 10^−6^, and 0.05, respectively.

**Figure 3:**
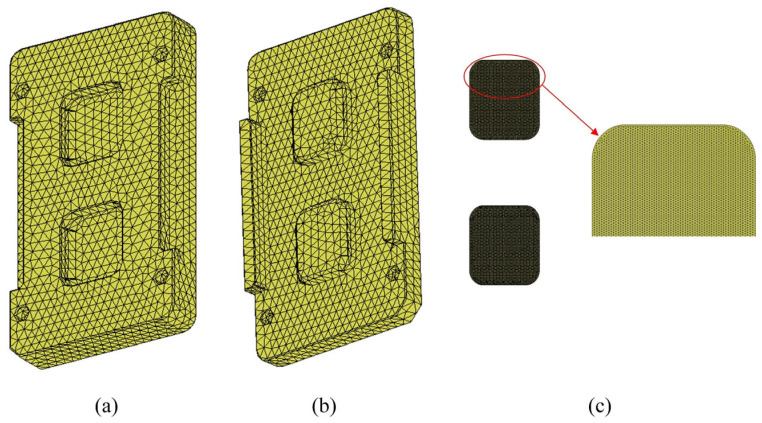
Finite element mesh: (**a**) upper mold; (**b**) lower mold; (**c**) glass components and zoomed in view.

To ensure the accuracy of the simulation, meticulous refinement of the mesh for both the glass and the forming areas of the mold was undertaken. As illustrated in Figure 3, the meshed model comprises 43,271 elements for the mold and 121,721 elements for the glass. Such a fine mesh is crucial for capturing the intricate interactions between the mold and the glass, thereby enhancing the validity and applicability of the simulation results in real-world manufacturing scenarios.

### 2.4. Experimental Equipment

**Figure 4:**
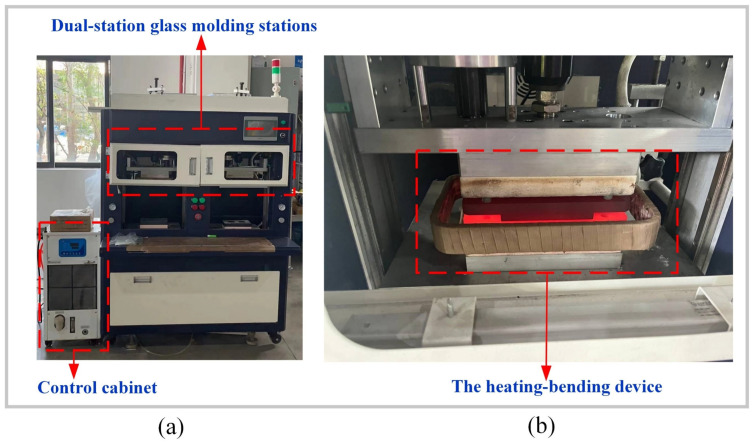
Three-dimensional ultra-thin glass hot bending machine: (**a**) dual-station glass molding stations; (**b**) heating systems.

A dual-station molding machine (manufactured by the Intelligent Robotics Institute of Guangdong Province) was used for the glass component molding trials in this study, as depicted in Figure 4a. This equipment features a dual-station design, allowing simultaneous processing tasks at two stations within one cycle, thereby significantly enhancing production efficiency. The primary parts of a dual-station molding machine typically include mold heating systems, glass feeding systems, molding systems, cooling systems, and demolding systems. During operation, glass raw materials are first preheated and softened in the heating system, then fed into the molding system for shaping, as illustrated in Figure 4b. The molded glass products undergo rapid cooling in the cooling system to maintain shape stability. Finally, the products are removed from the molds using the demolding system, producing glass components. The primary advantage of this dual-station design lies in its ability to perform cooling and demolding operations at one station while molding operations are conducted at the other. This alternating operation enables continuous production, significantly boosting equipment productivity and efficiency.

### 2.5. Scheme Design

Based on previous research experience and the relevant literature [21,35], the L_16_(4⁵) orthogonal design has demonstrated relatively good performance. This chapter employed an L_16_(4^5^) orthogonal array for simulation testing, assigning values to five key factors: heating rate (*A*), pressure frequency (*B*), forming temperature (*C*), forming pressure (*D*), and cooling rate (*E*), as shown in Table 3. In this study, the term forming pressure refers to the working pressure of the cylinder rather than the actual pressure applied to the glass surface. These control variables were set based on a thorough consideration of real-world conditions and an analysis of previous research findings. Through integrated use of finite element analysis and orthogonal experimental design methods, 16 data samples were analyzed, encompassing simulated predictions of glass component performance under different combinations of control variables. Table 3 and Table 4 share the same variables, with Table 4 provides a detailed presentation of each sample’s performance on quality attributes such as residual stress and shape deviation, along with corresponding energy efficiency data.

## 3. Simulation Results and Analysis

During the manufacturing process of glass components, molding parameters play a crucial role in determining both energy efficiency and product quality. By adjusting parameters such as temperature, pressure, and cooling rate, the molding process can be further optimized to enhance energy efficiency while ensuring high-quality outputs. This optimization not only reduces energy consumption but also minimizes defects and improves the overall performance and longevity of molded products.

### 3.1. Heating Process

**Figure 5:**
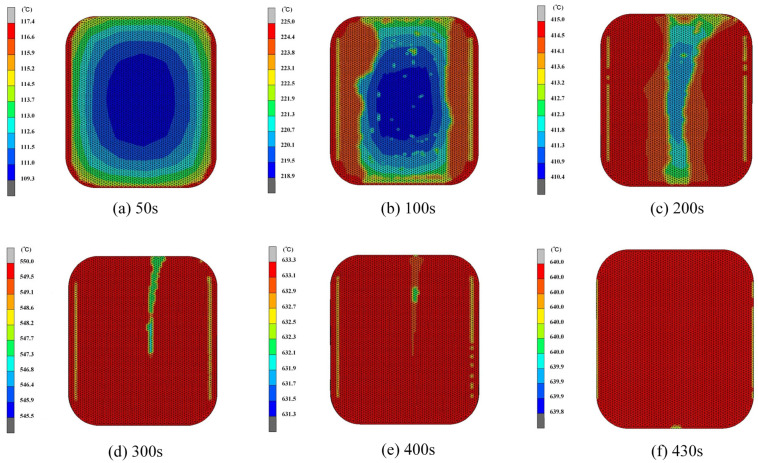
Temperature distribution of the ultrathin glass component during heating and equilibration phases: (**a**) 50 s; (**b**) 100 s; (**c**) 200 s; (**d**) 300 s; (**e**) 400 s; (**f**) 430 s (Setting parameters: heating rate = 1.5 °C/s, holding time = 80 s, forming temperature = 640 °C, forming pressure = 0.30 MPa, cooling rate = 1 °C/s).

The distribution of temperatures at different heating stages of the smartwatch’s ultra-thin glass components, as shown in Figure 5, revealing a dynamic trend where prolonged heating time results in varying surface temperatures of the glass component. the glass component’s surface temperature differential during the initial heating stage can reach 8.1 °C, as shown in Figure 5a. As the primary heat source, contact heat conduction from the bottom mold is mostly to blame for this problem, causing the temperature at the central part of the glass to be higher than that at the edges. This temperature variation is mainly because the mold does not directly contact the edges of the glass. Throughout the 300 s heating process, as the furnace temperature gradually increases, the glass component’s temperature differential drops to about 5 °C, as shown in Figure 5d, indicating that with continued heating, the temperature distribution of the glass components becomes more uniform. The procedure advances to the soaking stage, when the temperature rises to the necessary 640 °C forming temperature, as shown in Figure 5f.

**Figure 6:**
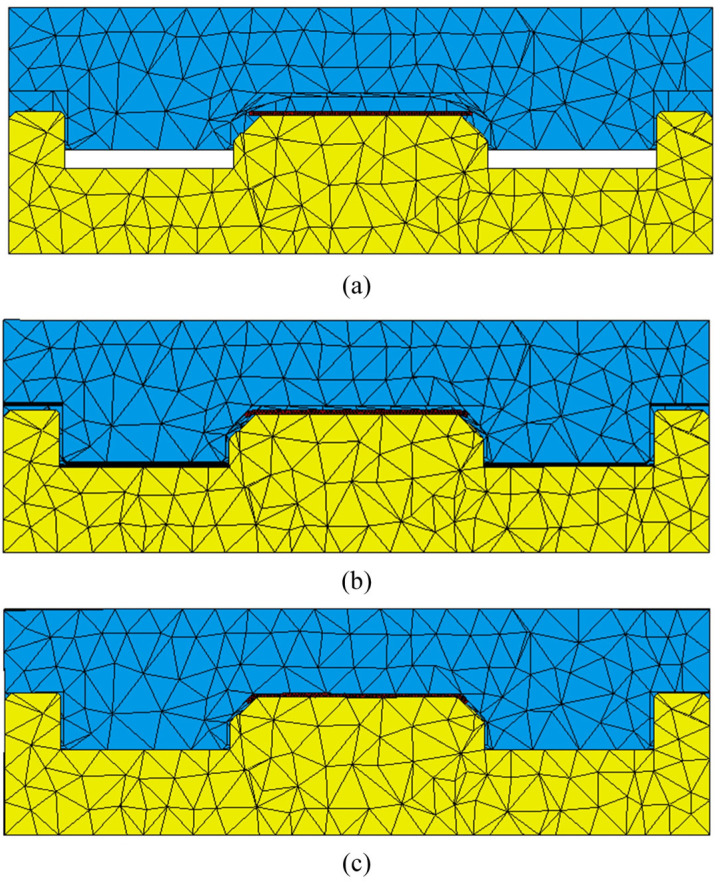
Glass deformation and flow process: (**a**) before deformation; (**b**) during deformation, and (**c**) after deformation.

The viscoelastic glass starts to distort when the mold pressure reaches the necessary temperature, as illustrated by the flow and deformation shown in Figure 6a. Initially, there is a 2 mm gap between the glass and the upper mold, with only partial contact with the lower mold, indicating the impending deformation. As the upper mold further compresses, the viscoelastic glass starts to deform under the mold pressure, gradually extending from the center to the edges. After the upper mold descends by 1.5 mm, Figure 6b illustrates the glass’s flow condition and deformation, the glass component’s molding process is finished in Figure 6c.

### 3.2. Residual Stress of Glass Components

The main parameters affecting residual stress in the product are detailed in Table 5. The three parameters that have the biggest effects on residual stress in the glass component are cooling rate (*E*), forming temperature (*C*), and forming pressure (*D*), according to the data in Table 5.

**Figure 7:**
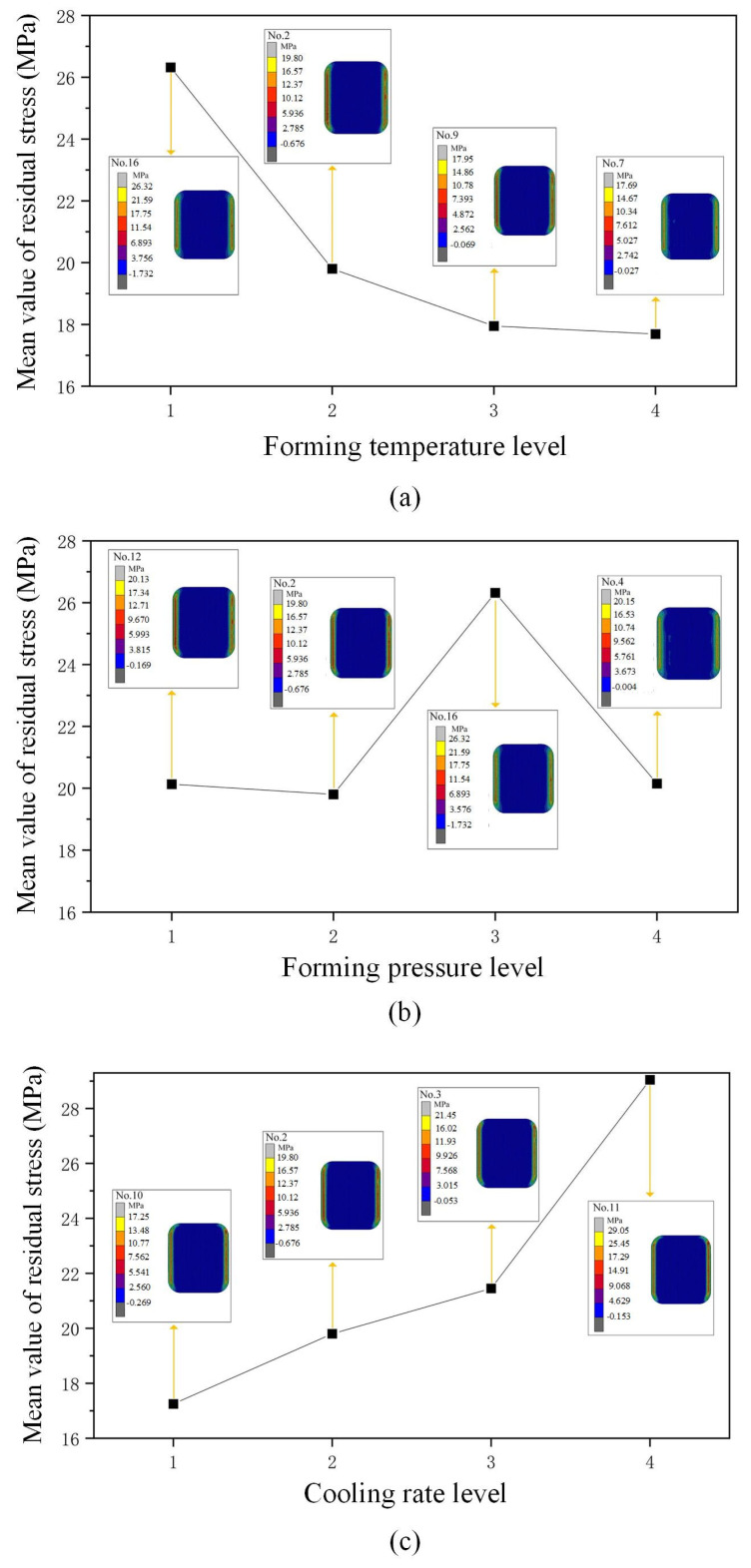
Residual stress main parameters effects: (**a**) forming temperature; (**b**) forming pressure; (**c**) cooling rate.

There is a noticeable reduction in residual stress in the glass component as the forming temperature is gradually raised, as shown in Figure 7a. Each level has four sets of data, and the most representative maximum residual stress is taken from one of these four sets. The glass component’s highest residual stress is 26.30 MPa when the forming temperature is 610 °C (No. 16,). However, increasing the forming temperature to 630 °C (No. 9) significantly reduces the maximum residual stress to 17.95 MPa, and further increasing it to 640 °C (No. 7) results in a further decrease to 17.60 MPa.

This trend can be elucidated by the viscoelastic properties of glass. The glass becomes less viscous as the forming temperature rises, which in turn enhances its rheological properties. This improvement in rheological behavior facilitates the flow and deformation of the glass during the forming process, contributing to a reduction in internal stress. Consequently, the maximum residual stress within the formed glass product is significantly lowered. These changes in physical properties underscore the importance of carefully controlling the forming temperature to optimize the quality and structural integrity of glass components.

From Figure 7b, the influence of forming pressure on the glass component’s maximum residual stress can be observed. With the forming pressure increases from 0.20 MPa (No. 12) to 0.30 MPa (No. 16), the glass component’s maximum residual has increased noticeably, rising from 20.10 MPa to 26.30 MPa—an increase of approximately 1.3 times.

This increase is attributed to the accelerated flow and deformation of softened glass due to the increased forming pressure, leading to shorter forming times for the glass component. However, within the shorter deformation time, internal equilibrium adjustments within the glass may still be incomplete, resulting in minimal stress release and thus an increase in the maximum residual stress value. It is noteworthy, however, that an anomaly is observed in Figure 7b (No. 4). As forming pressure increases, the maximum residual stress value does not continue to rise but instead shows a decreasing trend. This anomaly may be due to plastic deformation of the glass material with increasing forming pressure, which to some extent helps reduce residual stress.

From Figure 7c, the cooling rate greatly impacts the glass component’s maximum residual stress. Specifically, at a slower cooling rate of 0.25 °C/s (No. 10), the maximum residual stress within the glass measures 17.25 MPa, whereas with an increase in cooling rate to 1 °C/s (No. 11), the maximum residual stress shows a notable increase, rising to 29.00 MPa, demonstrating an approximately linear growth trend.

When the glass surface cools more rapidly, the surface layer contracts first and is constrained by the internal material, resulting in tensile stress. Meanwhile, the internal material, still at a higher temperature and constrained by the surface layer, remains in an expanded state, generating compressive stress, which exacerbates the accumulation of residual stress.

### 3.3. Shape Deviation of Glass Components

**Figure 8:**
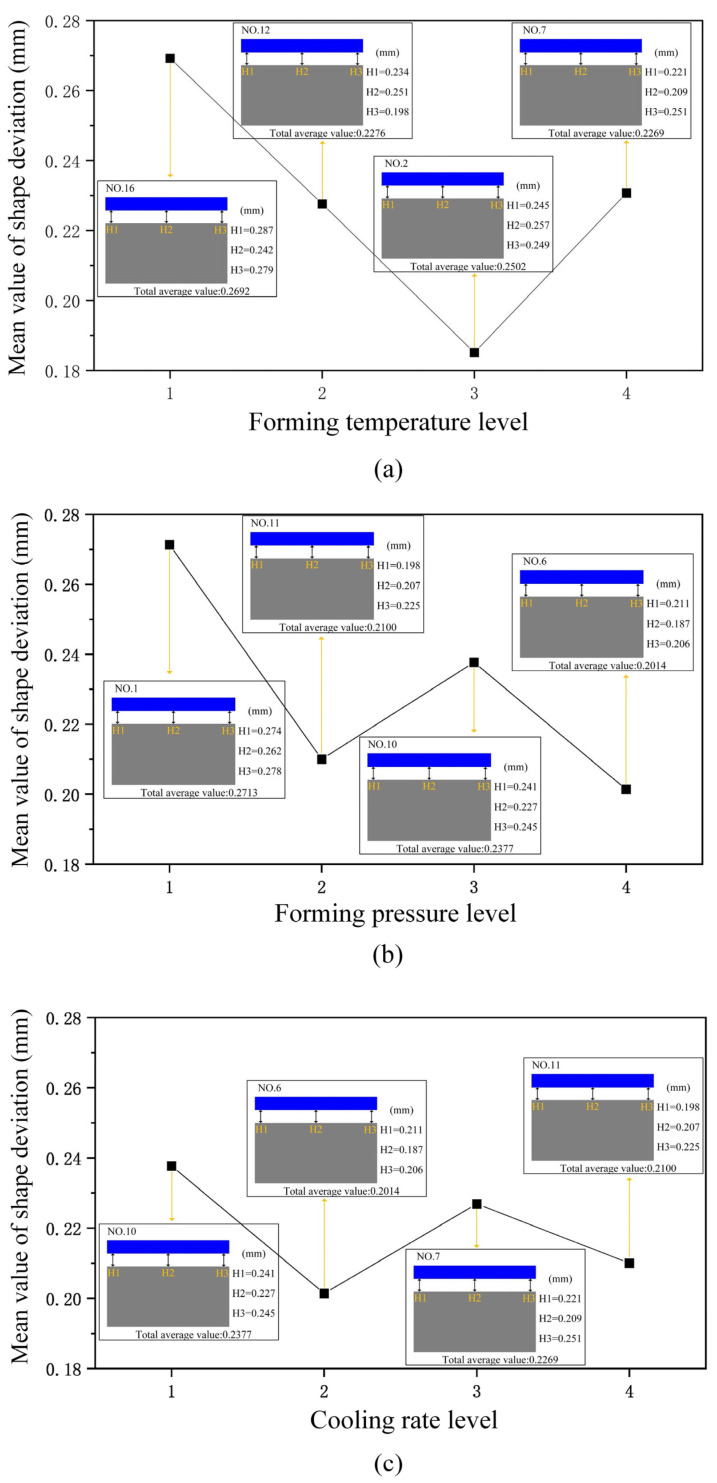
Shape deviations main parameters effects: (**a**) forming temperature; (**b**) forming pressure; (**c**) cooling rate.

Table 6 presents average response data on shape deviations of glass components, where forming temperature (*C*), forming pressure (*D*), and cooling rate (*E*) are identified as significant factors influencing product shape variations. Each level has four sets of data, and the most representative shape deviation is taken from one of these four sets. Further examination of Figure 8a reveals a trend in the relationship between shape deviation and forming temperature, characterized by an initial decrease followed by an increase. A linear decrease in shape deviation is observed as forming temperature increases from 610 °C (No. 16) to 620 °C (No. 2).

This decrease occurs because higher forming temperatures improve the glass’s rheological properties and enhance its fillability, thereby achieving a closer fit between the glass and the mold and reducing product shape deviations. However, as the temperature continues to rise to 640 °C (No. 7), shape deviation gradually increases. This is attributed to excessive deformation of the glass at higher temperatures, coupled with increased shrinkage upon cooling, leading to greater product shape deviations, likely caused by the glass’s excessive softening and flow at high temperatures.

Figure 8b illustrates the impact of glass component’s forming pressure on shape deviations of glass components. From Figure 8b, it is evident that the glass component’s forming pressure inversely affects shape deviations: at a pressure of 0.20 MPa (No. 1), the maximum shape deviation is 0.2713 mm, whereas increasing the forming pressure to 0.35 MPa (No. 6) reduces shape deviation by 0.0699 mm.

Higher forming pressures facilitate improved glass filling into the mold, enhancing the fit between the glass and the mold and thereby reducing shape deviations. Specifically, increased forming pressure improves the flowability of the glass, enabling a more uniform distribution within the mold, which results in more precise and accurate product shapes. This optimization of forming pressure not only ensures a better geometric conformity of the glass component but also contributes to the overall quality and consistency of the final product.

From Figure 8c, it can be observed that with increasing cooling rate, the glass component’s shape deviation processed initially rises and then decreases, with the inflection point occurring at a cooling rate of 0.75 °C/s. This phenomenon can be theoretically explained as the shape deviation steadily reduces when the glass component’s cooling rate rises from 0.25 °C/s (No. 10) to 0.75 °C/s.

Higher cooling rates reduce temperature differentials between the surface and interior of the glass, thereby decreasing temperature gradients and reducing thermal stresses, which helps minimize shape deviations because of the glass material’s calming properties. In other words, an appropriate cooling rate can, to some extent, suppress deformation of the glass during cooling, thus reducing shape deviations. However, when the cooling rate continues to increase to 1 °C/s (No. 11), shape deviations begin to increase. Rapid cooling of the mold at high temperatures may induce shrinkage or distortion, which transfers to the glass components, resulting in noticeable increases in shape deviations.

### 3.4. Energy Efficiency of Glass Components

The energy efficiency during the glass molding process must be comprehensively considered. This comprises the heat that is taken in by the glass material and the mold, as well as the heat that is used up by the nitrogen that is molded. The quantification of heat contribution within the production cycle can be accurately described and calculated using Equations (1) and (2).

(1) $$E_{e}=\lambda (Q_{1}+Q_{2})=\lambda (\sum_{2}^{i=1}c_{i}m_{i}\Delta T+c_{3}vt\rho \Delta T\prime )$$

(2) $$\lambda (T)=2.01+3.1\times 10^{-5}T$$

where *E_e_* represents the energy efficiency (kJ/pcs); *λ* represents the coefficient of thermal loss, a temperature *T*-dependent function; *Q*_1_, *Q*_2_ represents the heat usage of the molds and glass blanks, as well as heat usage of nitrogen (kJ/pcs); *m_i_* represents the molds and glass blank’s mass (kg); *c*_1–3_ represents the mold, glass, and nitrogen’s specific heat capacities (J/(kg·°C)); Δ*T* represents the variations in the glass blank’s and the molds’ temperatures (°C); Δ*T*′ represents the variations in nitrogen temperature (°C); ν represents the flow rate of nitrogen (mL/s); *t* represents the injection time of nitrogen (s); ρ represents the density of nitrogen (kg/mm^3^).

**Figure 9:**
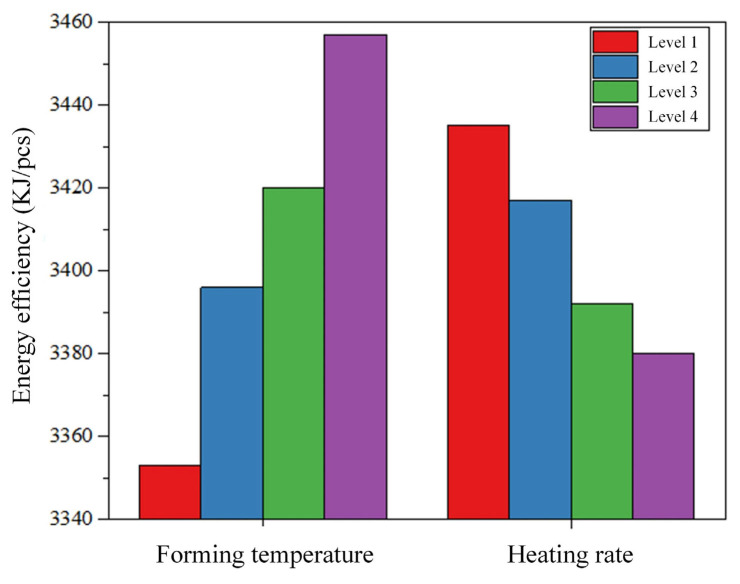
Effects of forming temperature and heating rate on energy efficiency in glass component production.

Table 7 presents the average response data for energy efficiency of glass components, with heating rate (*A*) and forming temperature (*C*) identified as significant elements affecting glass component energy efficiency. Further examination of Figure 9 reveals the relationship between these factors and energy efficiency. Producing glass components requires significantly more energy when the forming temperature rises, and higher temperatures may also enhance adhesion between the material and the mold, thereby increasing demolding difficulty.

Longer heating times are required to weaken this adhesion, making it easier for the material to release from the mold. This is because a faster heating rate reduces heating time, thereby reducing the product manufacturing cycle and heat loss from the furnace. Conversely, a decrease in heating rate and longer heating times in GMP lead to extended production cycles, increased heat loss, and higher energy efficiency. A slower heating rate means more time is needed to reach the glass softening point from the initial temperature, and since effective molding of glass requires specific temperatures, the overall heating stage is prolonged, thereby extending the overall production cycle. Prolonged heating also exacerbates temperature differentials between the mold and the surrounding environment, intensifying heat loss through convection and radiation. With extended heating times, equipment consumes more energy to maintain the required heating temperatures.

## 4. Process Optimization for Molding

### 4.1. Regression Analysis-Based Model

During the GMP process, variations in various control factors lead to significant changes in the target response function, and this nonlinear relationship makes it difficult to derive an exact analytical model directly. To effectively address this challenge, a regression analysis-based approach can be employed, integrating control factors into a statistical response model as represented by Equation (3). This method enables a more precise exploration of how each control factor influences the target response function, facilitating optimization of the GMP process to achieve improved product quality and energy efficiency performance.

(3) $$y(x)=c_{0}+\sum_{i=1}^{n}c_{i}x_{i}+\sum_{ij(i<j)}^{n}c_{ij}x_{i}x_{j}$$

where *y* represents the response function; *c*_0_, *c_i_*, and *c_j_*_,_ respectively denote the zeroth, first, and second-order coefficients; *x_i_* and *x_j_* represent control elements; and *n* is the number of factors.

Regression models for glass component’s residual stress (*R_s_*), shape deviation (*S_d_*), and energy efficiency (*E_e_*) were created using the commercial data analysis program Minitab 17. These models are shown by Equations (4)–(6).

(4) $$\begin{array}{l} R_{s}=-5292-96.1A-13.04B+20.76C-14.54D+89.0E-26.91A^{2}\\ -0.0047B^{2}-0.01902C^{2}-0.05521D^{2}-57.3E^{2}+0.908AB+0.301AC\\ +0.512AD+0.4255BD \end{array}$$

(5) $$\begin{array}{l} S_{d}=35.2+0.595A+0.0195B0.1295C+0.05D-0.29E+0.087A^{2}\\ -0.000005B^{2}+0.000119C^{2}+0.000101D^{2}+0.188E^{2}+0.00495AB\\ -0.00189AC-0.00455AD-0.00118 \end{array}$$

(6) $$\begin{array}{l} E_{e}=5640-671A-180B-1.4C+16.0D+169E+6.2A^{2}-0.079B^{2}-0.00\\ 71C^{2}-0.280D^{2}-112E^{2}+6.16AB+0.66AC+0.10AD+0.308BC \end{array}$$

### 4.2. Multi-Objective Optimization

The optimization method that forms the basis of NSGA-II is the NSGA algorithm, which Deb and Srinivas co-developed [36]. NSGA-II significantly enhances the efficiency and accuracy of genetic algorithms through a swift and precise non-dominated sorting process, operating based on the Pareto optimality principle and closely integrating with elitist strategies, particularly excelling in optimizing structural model parameters [37]. This algorithm can simultaneously consider multiple objective functions, finding balanced trade-offs among them to achieve superior performance and outcomes. Further refining the precision of solutions, NSGA-II employs elitist strategies and a crowding comparison operator [38]. The algorithm integrates density considerations using the crowding distance comparison operator when selecting new generations, ensuring that specific populations are not marginalized and achieving a more balanced distribution of non-dominated solutions across the entire solution space. To modify the thermal bending properties of ultra-thin glass for 3D designs of smartwatches, the NSGA-II optimization approach was utilized in this study. This approach not only significantly reduced the computational time and costs but also effectively maintained population diversity, effectively reducing the chance of reaching local optima through convergence.

To reduce glass component’s shape deviation, residual stress, and energy efficiency, multi-objective optimization was carried out on the glass component’s molding parameters using NSGA-II. The setting of the operational parameters is as follows:

(1) *f*_min_ = {*R_s_*, *S_d_*, *E_e_*};
(2) Population size = 100;
(3) Iteration number = 500;
(4) Stopped iterations = 500;
(5) Value deviation of the fitness function = 1 × e^−100^;
(6) Probability of crossover = 0.5;
(7) Probability of mutation = 0.0005.

**Figure 10:**
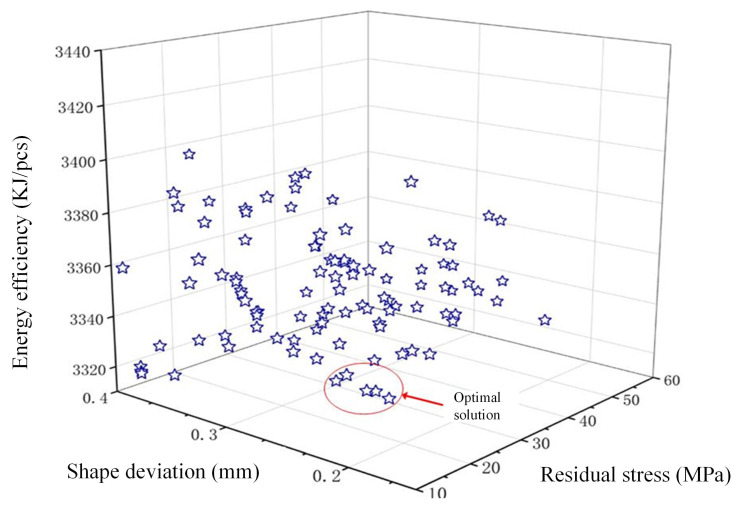
Pareto front for residual stress and shape deviation.

The challenge of attaining ideal states for glass component’s residual stress, shape deviation, and energy efficiency all at once is clearly illustrated in Figure 10. Therefore, in the context of multi-objective optimization, efforts are directed towards seeking the best balance among these three indicators. To minimize energy efficiency and reduce enterprise costs maximally, effective value management within acceptable ranges is crucial. The GMP solution proposed in this study can effectively reduce energy efficiency while ensuring product quality, achieving an ideal balance according to practical needs. As shown in Table 8, optimization of Pareto front solutions successfully improved these three objectives.

### 4.3. Experimental Validation

To validate the effectiveness of Pareto front solutions in optimizing the residual stress, shape deviation, and energy efficiency of glass components, various combinations of control parameters were employed in simulations and experiments. For each of the three goals, partial Pareto front solutions were produced through the optimization procedure. Numerical simulation experiments were conducted on the sets of solutions from the Pareto front belonging to Groups 2, 3, and 4. By comparing these simulation results with the selected Pareto front solutions, a more precise evaluation of the optimization effects was achieved.

**Figure 11:**
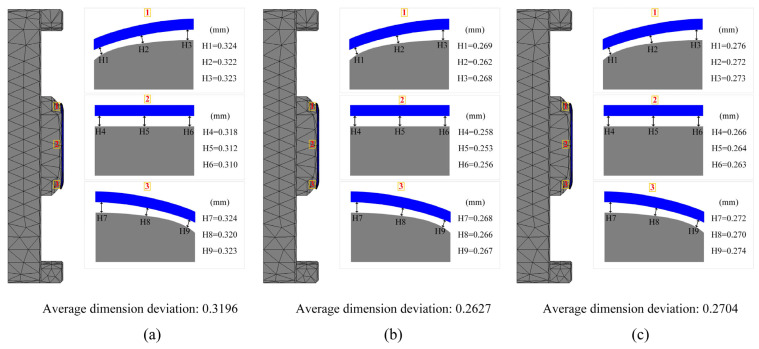
Shape deviation of glass components: (**a**) Group 2; (**b**) Group 3; (**c**) Group 4.

**Figure 12:**
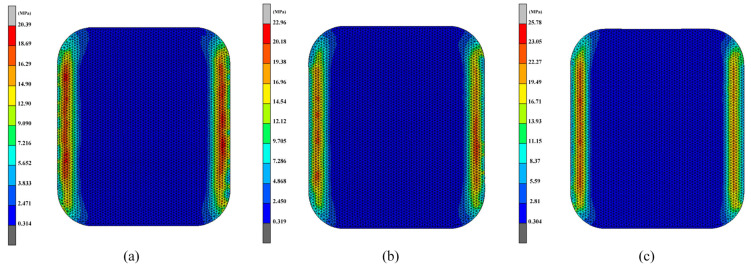
Residual stress of glass components: (**a**) Group 2; (**b**) Group 3; (**c**) Group 4.

Figure 11 and Figure 12 present the simulation results for glass component’s shape deviation and maximum residual stress. The simulated glass component’s shape deviation, maximum residual stress, and energy efficiency values for the set of data in Group 2 were 0.3196 mm, 20.39 MPa, and 3348.7 kJ/pcs, respectively. The shape deviation, maximum residual stress, and energy efficiency relative errors were determined to be 8.5%, 13.2%, and 2.9%, respectively, when compared to the optimum anticipated values. Similarly, the comparison of the sets of simulation results in Group 3 and Group 4 with the optimized values showed relative errors for shape deviation of 4.6% and 3.3%, maximum residual stress errors of 8.1% and 16.2%, and energy efficiency errors of 2.6% and 7.3%, respectively. Overall, the optimization predictions and simulation data exhibited good consistency, with all relative errors remaining within 20%, indicating significant optimization effects and accurate predictions. Table 9 displays the comprehensive comparison analysis findings, demonstrating that all discrepancies are within acceptable standards.

## 5. Discussion

Experimental validation showed that the average glass component’s shape deviation of the three groups samples presented in Table 9 reached 0.284 mm, with all errors within 20% of the simulation predictions. Because of the inherent limits of simulation software, errors in traditional simulation modeling are regarded as typical and challenging to eliminate. For instance, in the simulation of curved screen components for smartphones, He et al. [23] observed a maximum prediction curved screen component’s error of 17.62%. Chen et al. [27] observed an average shape deviation of 0.1380 mm in their study of large automotive glass components, with errors remaining below 20% compared to the simulation data. Yang et al. [39] studied the shape deviations and cracks in the molding process of curved ultra-thin glass. The simulated shape deviation was generally 0.0751 mm smaller than the corresponding experimental values, but overall trends aligned with variations in process parameters, thereby validating the model’s reliability.

Research on small glass components is relatively scarce, especially regarding the molding technologies used for smartwatches. This area of study is crucial given the precision and miniaturization needed for smartwatch components, which demand highly specialized molding techniques to ensure both quality and performance. Furthermore, in this study, the actual production shape deviation range for smartwatch glass components is approximately ±0.15 mm, while the predicted error margin based on the simulation model is around 0.2 mm. Considering the potential variables during the experimental process and the inherent confines of simulation modeling technology, the predicted error level in this study being below 20% is deemed acceptable.

Beyond its application in smart wearable devices, the integration of glass hot-pressing technology into hydraulic machinery has demonstrated considerable potential. This process enables precise three-dimensional shaping of glass in its viscoelastic state and may serve as an alternative fabrication route for components requiring high optical transmittance, compressive strength, and corrosion resistance. In recent years, the demand for high-precision transparent observation windows, corrosion-resistant flow monitoring elements, and complex streamlined flow-guiding structures has continued to rise. However, traditional glass processing methods have been constrained by the material’s inherent brittleness and limited forming accuracy, making it difficult to satisfy engineering demands under complex service conditions. In contrast, hot-pressing allows for high-fidelity replication of three-dimensional geometries through precise control of temperature and pressure, offering a critical technological pathway for the fabrication of advanced functional glass components. Similarly to recent advances in laser-induced plasma micro-machining of CFRP materials [40], which highlight the benefits of fine energy control and liquid-assisted processing in reducing material damage, hot-pressing offers comparable precision and control advantages in glass shaping applications.

## 6. Conclusions

This study employed a multivariable experimental design methodology to successfully construct an accurate molding model for smartwatch glass components. Subsequently, the effective NSGA-II multi-objective optimization method was then used to accomplish the goal of synergistic optimization between quality features and energy efficiency. The following is a summary of the primary findings:

(1) A detailed simulation model of smartwatch glass component molding was established, simulating the heat transfer characteristics of the molding system. The results indicate that the maximum shape deviation occurs at the center of the glass component. Additionally, it was found that the primary concentration point of stress distribution lies in the curved deformation area of the molded component.
(2) Under conditions of forming temperature at 630 °C, forming pressure at 0.25 MPa, and cooling rate at 0.25 °C/s, the residual stress in glass products is relatively low. Maintaining the forming temperature at 630 °C, increasing the forming pressure to 0.30MPa, and raising the cooling rate to 0.75 °C/s minimize shape deviation. Additionally, when the forming temperature of the glass component is 610 °C and the heating rate is 3 °C/s, the production energy efficiency during production is relatively low.
(3) An analysis of a synergistic balance scheme between quality features and energy efficiency was conducted using the NSGA-II tri-objective optimization. With a maximum error of no more than 20%, which is within an acceptable range, the optimized projected results demonstrated good agreement with the simulation and experimental findings.

## Figures and Tables

**Table 1:** Mechanical and thermal properties of G-11 glass and graphite materials [29].

Properties	Glass Material	Graphite Material
Young’s modulus E (GPa)	72.6	10.2
Poisson’s ratio v	0.206	0.25
Density ρ (g/cm^3^)	2.51	1.78
Thermal conductivity K(W/m·K)	1.1	151
Specific heat Cp (J/kg·°C)	858	720
Thermal expansion coefficient (°C^−1^)	Liquid 3.43 × 10^−5^ Solid 1.143 × 10^−5^	4.8 × 10^−6^

**Table 3:** Control factors and standard settings of levels.

No.	Control Factors				
	*A* (°C/s)	*B* (Hz)	*C* (°C)	*D* (MPa)	*E* (°C/s)
1	1.5	0	610	0.20	0.25
2	2	10	620	0.25	0.5
3	2.5	30	630	0.30	0.75
4	3	50	640	0.35	1

**Table 4:** Experimental design and response statistics.

No.	Control Factors					Residual Stress (MPa)	Shape Deviation (mm)	Energy Efficiency (kJ/pcs)
	*A* (°C/s)	*B* (Hz)	*C* (°C)	*D* (MPa)	*E* (°C/s)			
1	1.5	0	610	0.20	0.25	18.65	0.2713	3365
2	1.5	10	620	0.25	0.5	19.80	0.2502	3432
3	1.5	30	630	0.30	0.75	21.45	0.1851	3450
4	1.5	50	640	0.35	1	20.15	0.2307	3493
5	2	0	620	0.30	1	28.72	0.1913	3408
6	2	10	610	0.35	0.75	28.58	0.2014	3365
7	2	30	640	0.20	0.5	17.69	0.2269	3469
8	2	50	630	0.25	0.25	15.21	0.2157	3426
9	2.5	0	630	0.35	0.5	17.95	0.1928	3401
10	2.5	10	640	0.30	0.25	17.25	0.2377	3444
11	2.5	30	610	0.25	1	29.05	0.2100	3341
12	2.5	50	620	0.20	0.75	20.13	0.2276	3383
13	3	0	640	0.25	1	20.85	0.1868	3420
14	3	10	630	0.20	0.75	21.18	0.2169	3401
15	3	30	620	0.35	0.25	18.84	0.2210	3359
16	3	50	610	0.30	0.5	26.32	0.2692	3341

**Table 2:** Stress relaxation and structural relaxation parameters of G-11 glass [28,29].

Stress Relaxation		Structural Relaxation	
Shear Modulus (MPa)	Relaxation Times (s)	Weight Coefficient	Relaxation Times (s)
12,566	0.0689	0.108	3.0
		0.443	0.671
12,615	0.0065	0.166	0.247
		0.161	0.091
4582	0.0001	0.046	0.033
		0.077	0.008

**Table 5:** Mean residual table for stress response.

Level	*A*	*B*	*C*	*D*	*E*
1	20.01	21.53	25.61	19.36	17.47
2	22.50	21.66	21.86	21.20	20.41
3	21.08	21.71	18.92	23.43	22.79
4	21.75	20.44	18.95	21.35	24.67
Delta	2.49	1.27	6.69	4.07	7.19
Order	4	5	2	3	1

**Table 6:** The mean response table for shape deviations.

Level	*A*	*B*	*C*	*D*	*E*
1	0.2247	0.2009	0.2284	0.2280	0.2268
2	0.1992	0.2169	0.2129	0.2061	0.2252
3	0.2074	0.2011	0.1930	0.2112	0.1981
4	0.2138	0.2262	0.2109	0.2018	0.1951
Delta	0.0255	0.0252	0.0353	0.0262	0.0317
Order	4	5	1	3	2

**Table 7:** Mean effects table for energy efficiency.

Level	*A*	*B*	*C*	*D*	*E*
1	3435	3399	3353	3405	3399
2	3417	3411	3396	3405	3411
3	3392	3405	3420	3411	3400
4	3380	3411	3457	3405	3416
Delta	55	12	104	6	17
Order	2	4	1	5	3

**Table 8:** Pareto front solutions for multi-objective optimization of three targets.

No.	Control Factors					Residual Stress (MPa)	Shape Deviation (mm)	Energy Efficiency (kJ/pcs)
	*A* (°C/s)	*B* (Hz)	*C* (°C)	*D* (MPa)	*E* (°C/s)			
1	1.64	43.13	609.4	0.2256	0.65	18.7	0.2605	3367
2	2.78	36.90	617.7	0.3350	0.45	17.7	0.2924	3339
3	1.67	43.20	611.1	0.2331	0.63	21.1	0.2506	3382
4	1.72	43.12	610.0	0.2343	0.56	21.6	0.2615	3369
5	1.78	42.89	608.9	0.2311	0.60	23.1	0.2537	3364
6	1.68	43.18	623.1	0.2739	0.66	29.8	0.2359	3396
7	1.82	43.20	608.9	0.2422	0.47	22.3	0.2609	3358

**Table 9:** Optimization prediction results and the simulation results.

Group	Control Factors					Simulation Results			Relative Error		
	*A*	*B*	*C*	*D*	*E*	*R_s_* (MPa)	*S_d_* (mm)	*E_e_* (kJ/pcs)	*R_s_* (%)	*S_d_* (%)	*E_e_* (%)
2	2.78	36.90	617.7	0.3350	0.45	20.39	0.3196	3348.7	13.2	8.5	2.9
3	1.67	43.20	611.1	0.2331	0.63	22.96	0.2627	3472.3	8.1	4.6	2.6
4	1.72	43.12	610.0	0.2343	0.56	25.78	0.2704	3634.3	16.2	3.3	7.3

## Data Availability

Data are contained within the article as suggested.
